# Influence of labor on direct and indirect determinants of placental 11beta-hydroxysteroid dehydrogenase activity

**DOI:** 10.1007/s00404-020-05755-4

**Published:** 2020-09-03

**Authors:** Hanna Huebner, Kirsten Heussner, Matthias Ruebner, Matthias Schmid, Jennifer Nadal, Joachim Woelfle, Andrea Hartner, Carlos Menendez-Castro, Manfred Rauh, Matthias W. Beckmann, Sven Kehl, Fabian B. Fahlbusch

**Affiliations:** 1 Department of Gynecology and Obstetrics, University Hospital Erlangen, Comprehensive Cancer Center Erlangen-EMN, Friedrich-Alexander University Erlangen-Nuremberg, Erlangen, Germany; 2 Department of Pediatrics and Adolescent Medicine, Friedrich-Alexander-University of Erlangen-Nuremberg, Loschgestr. 15, 91054 Erlangen, Germany; 3 Institute of Medical Biometry, Informatics and Epidemiology (IMBIE), University Hospital of Bonn, Bonn, Germany

**Keywords:** LC–MS/MS, Placenta, Mode of delivery, CRH, 11beta-hydroxysteroid dehydrogenase

## Abstract

**Purpose:**

Labor is a complex process involving multiple para-, auto- and endocrine cascades. The interaction of cortisol, corticotropin-releasing hormone (CRH) and progesterone is essential. The action of cortisol on the human feto-placental unit is regulated by 11beta-hydroxysteroid dehydrogenase type 2 (11β-HSD2/HSD11B2) that converts cortisol into inactive cortisone. The majority of studies on the assessment of placental 11β-HSD2 function determined indirect activity parameters. It remains elusive if indirect measurements correlate with enzymatic function and if these parameters are affected by potential confounders (e.g., mode of delivery). Thus, we compared determinants of indirect 11β-HSD2 tissue activity with its direct enzymatic turnover rate in placental samples from spontaneous births and cesarean (C)-sections.

**Methods:**

Using LC–MS/MS, we determined CRH, cortisol, cortisone, progesterone and 17-hydroxy(OH)-progesterone in human term placentas (spontaneous birth vs. C-section, *n* = 5 each) and measured the enzymatic glucocorticoid conversion rates in placental microsomes. Expression of *HSD11B1, 2* and *CRH* was determined via qRT-PCR in the same samples.

**Results:**

Cortisol–cortisone ratio correlated with direct microsomal enzymatic turnover. While this observation seemed independent of sampling site, a strong influence of mode of delivery on tissue steroids was observed. The mRNA expression of *HSD11B2* correlated with indirect and direct cortisol turnover rates in C-section placentas only. In contrast to C-sections, CRH, cortisol and cortisone levels were significantly increased in placental samples following spontaneous birth.

**Conclusion:**

Labor involves a series of complex hormonal processes including activation of placental CRH and glucocorticoid metabolism. This has to be taken into account when selecting human cohorts for comparative analysis of placental steroids.

**Electronic supplementary material:**

The online version of this article (10.1007/s00404-020-05755-4) contains supplementary material, which is available to authorized users.

## Introduction

Labor strongly influences the human placental transcriptome [1–3] and post-transcriptional modifications like the phosphorylation of central signaling nexus, e.g., mammalian target of rapamycin (mTOR) [4]. It is further associated with progressive oxidative stress consistent with ischemia–reperfusion injury and induces the release of angiogenic, pro-inflammatory cytokines and pro-apoptotic factors in the human placenta [3]. So far, it remains elusive whether these labor-associated changes are a cause of labor onset and progress or simply an effect of labor, or both [1].

All of the above described placental processes are significantly influenced by the cross talk of the major endocrine regulators of labor, e.g., corticotropin-releasing hormone (CRH) and glucocorticoids, as well as progesterone and estradiol (E2) and are assumed to have relevant clinical impact for a healthy pregnancy [5, 6]. CRH is an important regulator of fetal growth via maintenance of placental glucose homeostasis. It further controls the timing of birth by influencing contractile properties of the myometrium via its interaction with progesterone and prostaglandin H2 synthase-2 [1, 7–11]. CRH levels rise during pregnancy and its expression increases significantly during labor [1]. CRH further controls fetal organ maturation by regulating placental 11beta-hydroxy-steroid dehydrogenase type 2 (11β-HSD2/HSD11B2) expression, and inducing fetal adrenocorticotropic hormone (ACTH) release [12]. This comprises a feedback loop that essentially depends on the rate of materno-fetal cortisol transport, which in turn is limited by the enzymatic activity of placental 11β-HSD2 [9] converting cortisol (F) to cortisone (E) and corticosterone (B) to dehydrocorticosterone (A).

However, while 11β-HSD2 and CRH are important regulators of the above endocrine feedback loops that lead to labor induction and fetal maturation, it yet remains unknown (1) as to what degree labor itself might influence placental cortisol metabolism, (2) how to reliably asses the activity of 11β-HSD2 in placental tissue and (3) how labor affects the CRH and cortisol relation. There is a multitude of previous studies (e.g., [13]), including our own [14, 15], that have assessed placental 11β-HSD2 activity by determining its mRNA expression levels. So far, it remains unknown, if different indirect measurements of 11β-HSD2 activity (i.e., qRT-PCR and tissue steroid levels) show comparable results and if these accurately reflect the direct enzymatic activity in the placenta. We have established an LC–MS/MS-based method for the analysis of 11β-HSD2 activity in rodent and human placental tissue [16, 17]. This method enables indirect (cortisol/cortisone ratio) and direct (in vitro measurement of cortisol conversion following tissue extraction of microsomal 11β-HSD2) determination of 11β-HSD2 activity. The use of LC–MS/MS shows advantages over classic (radiolabeled) immunoassays, as it improves sample handling, reduces matrix effects, allows for high-throughput analysis and offers the possibility of studying multiple steroids simultaneously [16]. Moreover it permits the concomitant quantification of placental CRH [16, 17]. Thus, we set out to analyze the relation of indirect and direct indicators of 11β-HSD2 activity under the influence of labor by means of qPCR and LC–MS/MS.

## Materials and methods

### Cohort

Five term placentas from elective (non-emergency) singleton Cesarean (C-) sections (breach positions, re-sections) before the onset of labor and five term placentas from singleton spontaneous births were collected immediately after birth. The range of gestational age was 37 + 5 to 40 + 3 weeks and the mean maternal age was 35.3 years. Detailed patient characteristics are given in Table 1. All participating mothers and their newborns were healthy. No pregnancy complication or history of gestational disease was present.

**Table 1:** Clinical characteristics of pregnancies by labor groups

	Spontaneous birth (*n* = 5)	Cesarean section (*n* = 5)
Maternal age in years median (range)	33 (27–37)	35 (30–38)
Sex (number female/male)	3/2	2/3
Placental weight in grams median (range)	500 (480–580)	520 (430–560)
Birth weight in grams median (range)	3400 (3100**–**3660)	3510 (2960**–**3620)
Length at birth in cm median (range)	51 (50–52)	55 (48–56)

### Ethics

All participants gave their written informed consent with the approval by the Ethics Committee of the University of Erlangen-Nuremberg (#2625 02/28/02). All procedures were carried out in accordance with The Code of Ethics of the World Medical Association (Declaration of Helsinki) for experiments involving humans.

### Sample collection

After removal of decidua and fetal membranes, placental tissue (~ 8 g per sample, mid-depth) was obtained from six non-calcified placental areas with increasing distance to the umbilical cord (central/medial/peripheral), as previously described [17, 18]. All collected samples were immediately snap-frozen in liquid nitrogen and stored at − 80 °C until further use.

### Placental tissue steroid profiling

Placental tissue steroid profiling (indirect determination of HSD11B-activity) and CRH measurement were performed applying our established LC–MS/MS method, as described by us in detail elsewhere [16, 17]. Fractions from each of the three placental samples (total of 3 × 5 samples per group) were used. In short, per 0.5 g of tissue, 1.02 ml of ethanol containing 20 µl/ml proteinase-inhibitor cocktail (COMPLETE, Roche Diagnostics Deutschland GmbH, Penzberg, Germany) was added. Tissues were homogenized at 4 °C using a Precellys^®^ Ceramic Kit on a Precellys^®^ 24 tissue grinder equipped with a Cryolys-module for liquid nitrogen cooling (Peqlab, Erlangen, Germany). Conditions were 6 × 30 s at 224×*g* with an inter-cycle pause of 40 s. Subsequently, samples were ultrasonicated on ice (UW2070, Bandelin Electronic, Berlin, Germany) (settings: cycle 5, power 50%, 40 s). The homogenized samples were transferred into Eppendorf LoBind tubes (Fisher Scientific GmbH, Schwerte, Germany). After centrifugation for 10 min (23,000×*g*, 4 °C), the supernatant was used for further analysis.

### Determination of placental tissue 11β-HSD2 activity

The direct analysis of 11β-HSD2-enzymatic activity required the removal of a ~ 2.5 g fraction from each of the three samples (total of 3 × 5 samples per group) for the extraction of microsomes. A detailed description of the used extraction method was published by Lakshmi and Monder [19] and involves the differential centrifugation of tissue lysates. 11β-HSD2 activity was measured by LC–MS/MS [16, 20].

### LC–MS/MS

LC–MS/MS analysis has been previously described by us [16, 17, 20]. In short, the autosampler was a CTC PAL-LC System (CTC Analytics, Zwingen, Switzerland), and for LC–MS/MS analysis a Triple-Quadrupol Mass spectrometer was used (API 4000 QTrap, Applied Biosystems, MDS SCIEX, Darmstadt, Germany). LC–MS/MS data analysis was performed using Analyst Software (Version 1.6.2, Applied Biosystems/MDS SCIEX, Darmstadt, Germany).

### RNA extraction and qRT-PCR analysis

RNA was extracted from placental tissues using peqGOLD TriFast (VWR, Darmstadt, Germany) and the chloroform–phenol extraction method [18]. RNA was treated with DNase I (Roche, Mannheim, Germany) and transcribed to cDNA using the High-Capacity-cDNA-Reverse-Transcription kit (Thermo Fisher, Darmstadt, Germany). Quantification of mRNA expression was performed by quantitative Realtime PCR (qRT-PCR) as previously described [21]. 40 ng of placental cDNA was used. Both 18srRNA and GAPDH expression was analyzed and used as housekeeping gene. qRT-PCR analysis was performed using the SYBR select master mix (Thermo Fisher, Darmstadt, Germany) The primer sequences were as follows: HSD11B2_fwd ACCAAACCAGGAGACATTAG, HSD11B2_rev TCAGCAACTATTCATTGTG, HSD11B1_fwd ACCACCTTCTGTAGAGTTTC, HSD11B1_rev AGAGAGATGGCTTATCATCTG, CRH_fwd CCGTTTCCAGGTGTTTATAG, CRH_rev AGATTTAGTCTTACCCACCC, 18srRNA_fwd AGATTTAGTCTTACCCACCC 18srRNA_rev GGCCTCACTAAACCATCCAA, GAPDH_fwd CTCTGCTGTAGGCTCATTTGC, GAPDH_rev ACCAAAGTTGTCATGGATGACCT.

### Statistical analysis

Statistical analysis was performed with SPSS^®^ Version 25 (SPSS Inc., Chicago, IL, USA). For normalization of data, measurements were log-transformed, as previously described [22]. All values are presented as mean ± SEM. For all parameters, the measurements (sampling location central, medial and peripheral) in the diagnostic subgroups (mode of delivery and sex of newborn) were compared using two-way ANOVA. Possible dependencies were also shown with the correlation analysis. The limit of significance was set at a *p* value of < 0.05.

## Results

**Fig. 1:**
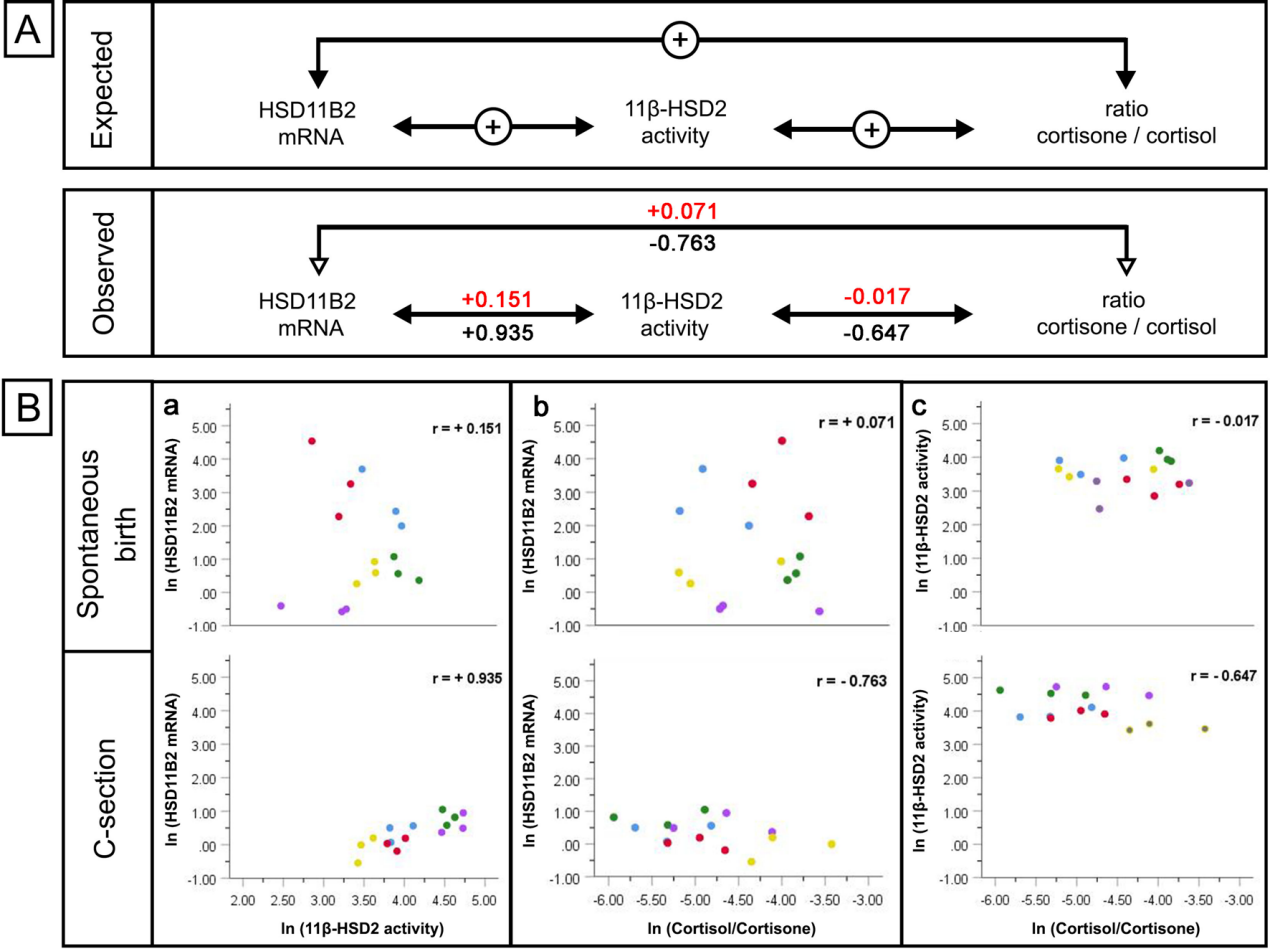
Pearson correlation of direct and indirect measures of HSD11B2 activity. **A** Expected and observed Pearson correlation coefficients are presented for correlation of *HSD11B2* mRNA expression (qRT-PCR), 11β-HSD2 activity directly measured by microsomal turnover and cortisol/cortisone ratios (LC–MS/MS). “+” represents a positive correlation, “−”a negative correlation. Pearson correlation coefficients of measures in placentas from spontaneous birth are marked red and those from cesarean section (C-section) black. **B** Dot blot diagrams are presented of spontaneous birth and C-section cases (*n* = 5 each), for measures of *HSD11B2* mRNA and activity levels (**a**), *HSD11B2* mRNA and cortisol/cortisone ratios (**b**) and 11β-HSD2activity and cortisol/cortisone ratios (**c**). Three placental samples per case were analyzed from different placental regions (central, medial, distal) and are represented by dots with matching color

We analyzed the correlation of direct (microsomal turnover) and indirect (qRT-PCR, glucocorticoid ratios) measures of 11β-HSD2 activity in placentas from spontaneous and C-section births (Suppl. Table 1). Of each patient, biopsies from the central (close to the umbilical cord), medial and peripheral area of the placenta were evaluated (Suppl. Table 2 and Suppl. Table 3). The results are displayed in Fig. 1. The expected correlation values are presented as “+” for positive correlation and “−” for negative correlation (Fig. 1a). As shown in Fig. 1a, should these indices of 11β-HSD2 activity be mutually utilizable, one would expect a positive correlation of *HSD11B2 mRNA* expression with 11β-HSD2 microsomal turnover rate and a negative correlation of these two parameters with the cortisol/cortisone ratio. The observed correlation coefficient for *HSD11B2* mRNA with 11β-HSD2 activity was 0.936 for the C-section cohort and 0.151 for the spontaneous birth cohort (Fig. 1a). Similarly, and in concordance with the expected correlation, the cortisol/cortisone ratio measured in placentas from C-sections correlated negatively with *HSD11B2* mRNA levels (*r* = − 0.743) and with the 11β-HSD2 activity (*r* = − 0.641, Fig. 1a, b). In contrast, no correlation between all measured parameters was detected in placentas from spontaneous delivery (Fig. 1a, b).

To further define whether potential confounders, i.e., mode of delivery, sex or sampling site, influenced direct or indirect measures of 11β-HSD2 activity, we performed an analysis of variance. The mode of delivery (Suppl. Table 1) had significant influence on both cortisol (*p* < 0.033) and cortisone (*p* < 0.009) levels, as well as on the ratio of the glucocorticoids corticosterone and dehydrocorticosterone (*p* < 0.016) (Table 2). In line with this finding, the mode of delivery had a significant influence on the placental turnover rate of dehydrocorticosterone by 11β-HSD1 (*p* < 0.037). Interestingly, the mode of delivery did not exert a significant influence on qPCR measurement of *CRH* and *HSD11B1* and *2*. While placental turnover of cortisol by 11β-HSD2 showed a trend (*p* < 0.067), 11β-HSD2 microsomal activity seemed generally unaffected by the mode of delivery (Table 2 and Suppl. Table 1).

**Table 2:** Log mean of steroid measurement

	Total	Mode of delivery				Sex of newborn			
		Spontaneous (*n* = 5); ln mean ± SEM	C-section (*n* = 5); ln mean ± SEM	*p* Value		Male (*n* = 5); ln mean ± SEM	Female (*n* = 5); ln mean ± SEM	*p* value	
				Medial-to-lateral	Mode of delivery			Medial-to-lateral	Sex of newborn
Tissue steroids									
Cortisol (F)*	− 4.45 ± 0.20	− 4.05 ± 0.24	− 4.86 ± 0.20	0.327	**0.033**	− 4.18 ± 0.31	− 4.73 ± 0.22	0.346	0.178
Cortisone (E)*	0.26 ± 0.07	+ 0.42 ± 0.05	+ 0.09 ± 0.08	0.656	**0.009**	0.22 ± 0.13	0.29 ± 0.07	0.746	0.670
Ratio F/E	− 4.71 ± 0.17	− 4.47 ± 0.20	− 4.95 ± 0.25	0.323	0.170	− 4.40 ± 0.24	− 5-02 ± 0.17	0.368	0.066
Corticosterone (B)*	− 5.67 ± 0.15	− 5.43 ± 0.24	− 5.91 ± 0.12	0.991	0.120	− 5.39 ± 0.22	− 5.95 ± 0.11	0.988	**0.050**
Dehydrocorticosterone (A)*	− 1.87 ± 0.17	− 1.47 ± 0.17	− 2.26 ± 0.15	0.685	**0.009**	− 1.95 ± 0.32	− 1-78 ± 0.16	0.661	0.648
Ratio B/A	3.31 ± 0.28	+ 3.92 ± 0.35	+ 2.70 ± 0.20	0.719	**0.016**^**§**^	3.12 ± 0.49	3.49 ± 0.29	0.726	0.533
CRH**	2.75 ± 0.19	+ 2.67 ± 0.29	+ 2.82 ± 0.27	0.872	0.718	2.62 ± 0.25	2.88 ± 0.30	0.865	0.537
Progesterone*	1.86 ± 0.07	+ 1.84 ± 0.15	+ 1.87 ± 0.03	0.202	0.877	1.77 ± 0.90	1.94 ± 0.11	0.190	0.278
17OH-Progesterone*	− 2.18 ± 0.13	− 2.32 ± 0.22	− 2.03 ± 0.12	0.497	0.286	− 2.05 ± 0.21	− 2.29 ± 0.15	0.472	0.396
Microsomal									
11β-HSD2 (F) turnover***	3.76 ± 0.18	+ 3.45 ± 0.21	+ 4.08 ± 0.22	0.658	0.067	3.81 ± 0.25	3.71 ± 0.27	0.646	0.802
11β-HSD2 (B) turnover***	3.43 ± 0 23	+ 3.09 ± 0.32	+ 3.79 ± 0.27	0.158	0.133	3.70 ± 0.35	3.17 ± 0.28	0.077	0.272
11β-HSD1 (E) turnover***	− 0.56 ± 0.21	+ 0.06 ± 0.27	− 0.17 ± 0.34	**0.039**	0.625	− 0.04 ± 0.38	− 0.07 ± 0.19	**0.027**	0.957
11β-HSD1 (A) turnover***	1.09 ± 0.12	+ 1.33 ± 0.14	+ 0.86 ± 0.13	0.060	**0.037**	1.23 ± 0.78	0.95 ± 0.17	**0.028**	0.273
qPCR									
*CRH* (mRNA)****	0.44 ± 0 78	+ 1.40 ± 1.45	+ 0.53 ± 0.42	0.892	0.236	0.92 ± 1.46	− 0.05 ± 0.70	0.884	0.565
*HSD11B1* (mRNA)****	2.43 ± 0.53	+ 2.81 ± 1.03	+ 2.05 ± 0.33	0.090	0.502	2.70 ± 0.85	2.15 ± 0.71	0.064	0.628
*HSD11B2* (mRNA)****	0.78 ± 0.38	+ 1.27 ± 0.71	+ 0.29 ± 0.17	0.402	0.217	0.86 ± 0 63	0 71 ± 0.51	0.361	0.858

*nmol/g(tissue); **ng/g(tissue); ***nmol/g (protein) * min^−1^; **** ratio relative to r18S; SEM, standard error of the mean, ln, logarithm, *p* values ≤ 0.05 are presented in bold, ^§^Greenhouse–Geisser correction

In our study, the sex of the newborn seemed to significantly influence corticosterone levels (*p* < 0.050), as determined by tissue LC–MS/MS (Table 2 and Suppl. Table 4). A significant sampling site-related influence was observed for 11β-HSD2 turnover of inactive to active glucocorticoids only (Table 2).

## Discussion

In the last decades, endocrine research has greatly contributed to the understanding of para- and autocrine events at the level of the placenta, thereby giving rise to obstetric treatment options to maintain pregnancy. Cortisol, CRH and progesterone were identified to be among the key hormonal players of labor and parturition [5]. So far, however, analysis of their action mainly involves the serum measurement of each hormone separately. To overcome this limitation, we have established an LC–MS/MS-based steroid hormone analysis and determination of CRH in human and rat placental tissue [17]. This high-throughput method offers a highly sensitive and specific detection of multiple hormones in a single probe using the same assay and facilitates the subsequent analysis of complex local hormone cascades.

Using microsomal enzymatic cortisol turnover as direct measurement for placental 11β-HSD2 activity, indirect (qRT-PCR, tissue steroid levels) determinants of placental glucocorticoid metabolism were compared with those direct measurements considering the influence of labor.

Our results show a relevant effect of labor on placental glucocorticoid metabolism, leading to perturbation of the correlations of direct and indirect parameters of 11β-HSD2 activity otherwise seen in placentas from C-sections before the onset of labor. Especially, cortisone and cortisol values were significantly influenced by labor. In comparison to placentas from C-sections, placental tissue from spontaneous births had significantly lower levels of cortisol and increased levels of cortisone. This emphasizes the impact of labor on placental cortisol metabolism and further underlines the importance of taking the mode of delivery into account when analyzing metabolic changes during birth. This goes in line with a former study analyzing maternal stress and placental function at birth by quantifying cortisol and cortisone concentrations [23]. The main finding of this study was an association of pregnancy-related anxiety and fetal cortisol exposure. This correlation, however, was not significant when stratified by delivery mode, which indicated that the mode of delivery can be a significant bias and has to be taken into account when analyzing metabolic changes [23]. Similarly, it was shown earlier that placental gene expression changes significantly during labor, which leads to a differential expression of over 351 genes when compared to C-section pregnancies [1]. This also included genes of the cortisol pathway and steroid metabolism [1, 24]. Moreover, labor is known to be a strong activator of stress-response signaling pathways found in the placenta [25]. In addition to our tissue-specific findings of labor-related steroid changes, the mode of delivery also seems to influence fetal steroid concentration in the umbilical blood [26]. For further studies, it could be of interest to analyze whether specific stress reducing interventions (e.g., mindfulness-based stress reduction) during pregnancy and labor have the potential to reduce activators of the placental stress-response signaling pathway [27].

Besides analysis of the labor-induced effect, we additionally evaluated the potential confounders’ sampling site and sex. However, statistical analysis did not identify sampling site or sex as relevant confounders in this context. Significant differences were observed solely for corticosterone (sex) and 11β-HSD1 turnover (sampling site). Sampling site is a well-known influencing factor on gene expression patterns and, thus, has to be evaluated carefully during tissue sampling [28, 29]. Even though there are many differentially expressed genes within the placental regions, the impact of placental sampling site on parameters of the glucocorticoid metabolism is largely unknown. However, in contrast to our findings, others have found sex as an important influencing factor on placental expression of, e.g., glucocorticoid receptors [30]. Glucocorticoid receptors, which were not studied by us, are essential for placental regulation of transcription of genes involved in placental and fetal development [31]. However, the impact of differential glucocorticoid receptor expression on, e.g., sex-dependent cortisol and birth weight is controversially discussed [32].

We further showed that measurement of direct 11β-HSD2 activity by LC–MS/MS seemed to be a more reliable method compared to qRT-PCR analysis regardless of the mode of delivery. Deviations from the mean of measured turnover rates were similar in both cohorts (spontaneous and C-section births), while especially mRNA levels showed higher deviations in placentas from spontaneous births compared to placental samples obtained from C-sections. Interestingly, even though many studies showed differential placental gene expression when comparing vaginal and C-section delivery, little is known about the inter-sample deviation [1, 33, 34]. Similarly, despite the fact that studies with optimal sample collection protocols have previously emphasized the relevance of birth mode for mRNA analysis, inter-study deviations of mRNA expression levels have not been evaluated [35]. Placentas subjected to vaginal delivery are exposed to mechanical compression and an intermittent reduction in maternal blood supply both caused by uterine contractions [35–37]. Especially, the latter influences metabolic measurements through generation of oxidative stress and activation of oxidative stress-related signaling cascades. In contrast to C-sections, spontaneous births vary in the frequency and duration of contractions and thus might result in higher differences of various parameters. Similarly, administration of supplemental oxygen or anesthesia could further influence gene expression and metabolic parameters. In addition, the magnitude of gene expression change was shown to be related to the length of labor [38].

The increased deviations in mRNA expression levels in spontaneous birth placentas might also be responsible for the perturbation of the correlations of direct and indirect parameters of 11β-HSD2. While in C-section placentas, both the levels of HSD11B2 mRNA expression and cortisone/cortisol ratios correlated with the HDS11B2 turnover rates as expected, those correlations could not be shown in placentas from spontaneous birth.

In summary, we were able to show that the mode of delivery strongly influences the interplay between direct and indirect determinants of placental 11β-HSD2 activity. However, our study has some limitations which have to be taken into account. First, due to the complexity of our method used for evaluating microsomal 11β-HSD2 turnover rates, the number of analyzed cases was quite low (*n* = 5 per cohort). As a consequence, a statistical comparison of the correlations was not suitable. Second, to elucidate the impact of vaginal delivery on the placental glucocorticoid metabolism, more clinical and molecular determinants would be of interest (e.g., expression of glucocorticoid receptor isoforms, hypoxia-associated factors, duration of contractions, supplemental oxygen or anesthesia).

## Electronic supplementary material

Below is the link to the electronic supplementary material.

Supplementary material 1 (DOCX 40 kb)

## Data Availability

The datasets used and/or analyzed during for the presented manuscript are available from the corresponding author on reasonable request.

## References

[1] Lee KJ, Shim SH, Kang KM, Kang JH, Park DY, Kim SH, Farina A, Shim SS, Cha DH (2010). Global gene expression changes induced in the human placenta during labor. Placenta.

[2] Lappas M, Rice GE (2009). Transcriptional regulation of the processes of human labour and delivery. Placenta.

[3] Cindrova-Davies T, Yung HW, Johns J, Spasic-Boskovic O, Korolchuk S, Jauniaux E, Burton GJ, Charnock-Jones DS (2007). Oxidative stress, gene expression, and protein changes induced in the human placenta during labor. Am J Pathol.

[4] Lager S, Aye IL, Gaccioli F, Ramirez VI, Jansson T, Powell TL (2014). Labor inhibits placental mechanistic target of rapamycin complex 1 signaling. Placenta.

[5] Kota SK, Gayatri K, Jammula S, Kota SK, Krishna SV, Meher LK, Modi KD (2013). Endocrinology of parturition. Indian J Endocrinol Metab.

[6] Kuon RJ, Voss P, Rath W (2019). Progesterone for the prevention of preterm birth—an update of evidence-based indications. Geburtshilfe Frauenheilkd.

[7] Jones SA, Challis JR (1990). Steroid, corticotrophin-releasing hormone, ACTH and prostaglandin interactions in the amnion and placenta of early pregnancy in man. J Endocrinol.

[8] Grammatopoulos DK, Hillhouse EW (1999). Role of corticotropin-releasing hormone in onset of labour. Lancet.

[9] Thomson M (2013). The physiological roles of placental corticotropin releasing hormone in pregnancy and childbirth. J Physiol Biochem.

[10] Smith R, Nicholson RC (2007). Corticotrophin releasing hormone and the timing of birth. Front Biosci.

[11] Jones SA, Brooks AN, Challis JR (1989). Steroids modulate corticotropin-releasing hormone production in human fetal membranes and placenta. J Clin Endocrinol Metab.

[12] Fahlbusch FB, Ruebner M, Volkert G, Offergeld R, Hartner A, Menendez-Castro C, Strick R, Rauh M, Rascher W, Dotsch J (2012). Corticotropin-releasing hormone stimulates expression of leptin, 11beta-HSD2 and syncytin-1 in primary human trophoblasts. Reproduct Biol Endocrinol.

[13] McTernan CL, Draper N, Nicholson H, Chalder SM, Driver P, Hewison M, Kilby MD, Stewart PM (2001). Reduced placental 11beta-hydroxysteroid dehydrogenase type 2 mRNA levels in human pregnancies complicated by intrauterine growth restriction: an analysis of possible mechanisms. J Clin Endocrinol Metab.

[14] Tzschoppe A, Fahlbusch F, Seidel J, Dorr HG, Rascher W, Goecke TW, Beckmann MW, Schild RL, Struwe E, Dotsch J (2011). Dexamethasone stimulates the expression of leptin and 11beta-HSD2 in primary human placental trophoblastic cells. Eur J Obstet Gynecol Reprod Biol.

[15] Tzschoppe A, Struwe E, Blessing H, Fahlbusch F, Liebhaber G, Dorr HG, Rauh M, Rascher W, Goecke TW, Schild RL, Schleussner E, Scheler C, Hubler A, Dahlem P, Dotsch J (2009). Placental 11beta-HSD2 gene expression at birth is inversely correlated with growth velocity in the first year of life after intrauterine growth restriction. Pediatr Res.

[16] Heussner K, Ruebner M, Huebner H, Rascher W, Menendez-Castro C, Hartner A, Fahlbusch FB, Rauh M (2016). Species differences of 11beta-hydroxysteroid dehydrogenase type 2 function in human and rat term placenta determined via LC-MS/MS. Placenta.

[17] Fahlbusch FB, Ruebner M, Rascher W, Rauh M (2013). Combined quantification of corticotropin-releasing hormone, cortisol-to-cortisone ratio and progesterone by liquid chromatography-Tandem mass spectrometry in placental tissue. Steroids.

[18] Ruebner M, Strissel PL, Ekici AB, Stiegler E, Dammer U, Goecke TW, Faschingbauer F, Fahlbusch FB, Beckmann MW, Strick R (2013). Reduced syncytin-1 expression levels in placental syndromes correlates with epigenetic hypermethylation of the ERVW-1 promoter region. PLoS ONE.

[19] Lakshmi V, Monder C (1988). Purification and characterization of the corticosteroid 11 beta-dehydrogenase component of the rat liver 11 beta-hydroxysteroid dehydrogenase complex. Endocrinology.

[20] Fahlbusch FB, Heussner K, Schmid M, Schild R, Ruebner M, Huebner H, Rascher W, Doerr HG, Rauh M (2015). Measurement of amniotic fluid steroids of midgestation via LC-MS/MS. J Steroid Biochem Mol Biol.

[21] Huebner H, Strick R, Wachter DL, Kehl S, Strissel PL, Schneider-Stock R, Hartner A, Rascher W, Horn LC, Beckmann MW, Ruebner M, Fahlbusch FB (2017). Hypermethylation and loss of retinoic acid receptor responder 1 expression in human choriocarcinoma. J Exp Clin Cancer Res.

[22] Tzschoppe A, Struwe E, Rascher W, Dorr HG, Schild RL, Goecke TW, Beckmann MW, Hofner B, Kratzsch J, Dotsch J (2011). Intrauterine growth restriction (IUGR) is associated with increased leptin synthesis and binding capability in neonates. Clin Endocrinol.

[23] Dahlerup BR, Egsmose EL, Siersma V, Mortensen EL, Hedegaard M, Knudsen LE, Mathiesen L (2018). Maternal stress and placental function, a study using questionnaires and biomarkers at birth. PLoS One.

[24] Sheehan PM, Bousman C, Komiti A, Judd F, Newman L, Tonge B, Castle D, Everall I (2019). Assessment of placental cortisol pathway gene expression in term pregnant women with anxiety. Neuropsychobiology.

[25] Burton GJ, Yung HW, Cindrova-Davies T, Charnock-Jones DS (2009). Placental endoplasmic reticulum stress and oxidative stress in the pathophysiology of unexplained intrauterine growth restriction and early onset preeclampsia. Placenta.

[26] Wynne-Edwards KE, Edwards HE, Hancock TM (2013). The human fetus preferentially secretes corticosterone, rather than cortisol, in response to intra-partum stressors. PLoS One.

[27] Lenz B, Eichler A, Schwenke E, Buchholz VN, Hartwig C, Moll GH, Reich K, Muhle C, Volz B, Titzmann A, Beckmann MW, Heinrich H, Kornhuber J, Fasching PA (2018). Mindfulness-based stress reduction in pregnancy: an app-based programme to improve the health of mothers and children (MINDFUL/PMI Study). Geburtshilfe Frauenheilkd.

[28] Wyatt SM, Kraus FT, Roh CR, Elchalal U, Nelson DM, Sadovsky Y (2005). The correlation between sampling site and gene expression in the term human placenta. Placenta.

[29] Mayhew TM (2008). Taking tissue samples from the placenta: an illustration of principles and strategies. Placenta.

[30] Saif Z, Hodyl NA, Hobbs E, Tuck AR, Butler MS, Osei-Kumah A, Clifton VL (2014). The human placenta expresses multiple glucocorticoid receptor isoforms that are altered by fetal sex, growth restriction and maternal asthma. Placenta.

[31] Bivol S, Owen SJ, Rose’Meyer RB (2016). Glucocorticoid-induced changes in glucocorticoid receptor mRNA and protein expression in the human placenta as a potential factor for altering fetal growth and development. Reprod Fertil Dev.

[32] Hodyl NA, Wyper H, Osei-Kumah A, Scott N, Murphy VE, Gibson P, Smith R, Clifton VL (2010). Sex-specific associations between cortisol and birth weight in pregnancies complicated by asthma are not due to differential glucocorticoid receptor expression. Thorax.

[33] Sober S, Reiman M, Kikas T, Rull K, Inno R, Vaas P, Teesalu P, Marti JML, Mattila P, Laan M (2015). Extensive shift in placental transcriptome profile in preeclampsia and placental origin of adverse pregnancy outcomes. Sci Rep.

[34] Peng HH, Kao CC, Chang SD, Chao AS, Chang YL, Wang CN, Cheng PJ, Lee YS, Wang TH, Wang HS (2011). The effects of labor on differential gene expression in parturient women, placentas, and fetuses at term pregnancy. Kaohsiung J Med Sci.

[35] Burton GJ, Sebire NJ, Myatt L, Tannetta D, Wang YL, Sadovsky Y, Staff AC, Redman CW (2014). Optimising sample collection for placental research. Placenta.

[36] Meizner I, Levy A, Katz M (1993). Assessment of uterine and umbilical artery velocimetry during latent and active phases of normal labor. Isr J Med Sci.

[37] Fleischer A, Anyaegbunam AA, Schulman H, Farmakides G, Randolph G (1987). Uterine and umbilical artery velocimetry during normal labor. Am J Obstet Gynecol.

[38] Rodriguez-Prado YM, Kong X, Fant ME (2013). PLAC1 expression decreases in chorionic villi in response to labor. ISRN Obstet Gynecol.

