# Supplementary material for: Influence of labor on direct and indirect determinants of placental 11beta-hydroxysteroid dehydrogenase activity
Source: Arch Gynecol Obstet. 2020 Sep 3;303(2):401–8. doi: 10.1007/s00404-020-05755-4 (PMC7858211; doi:10.1007/s00404-020-05755-4)
Supplement: Supplementary file 1 — Supplementary material 1 (DOCX 40 kb) [file 404_2020_5755_MOESM1_ESM.docx]

**Influence of Labor on Direct and Indirect Determinants of Placental 11beta-hydroxsteroid dehydrogenase Activity**

**Archives of Gynecology and Obstetrics**

Hanna Huebner, Kirsten Heussner, Matthias Ruebner, Matthias Schmid, Jennifer Nadal, Joachim Woelfle, Andrea Hartner, Carlos Menendez-Castro, Manfred Rauh, Matthias W. Beckmann, Sven Kehl, Fabian B. Fahlbusch^#^

**^#^corresponding author:** Fabian B. Fahlbusch

Address: Department of Pediatrics and Adolescent Medicine, Friedrich-Alexander-University Erlangen-Nürnberg, Loschgestr. 15, 91054 Erlangen, Germany

E-mail address: fabian.fahlbusch@uk-erlangen.de

Suppl. Table 1 Measurements by mode of delivery

|  | Mode of delivery | | | | | | | | | | | |
| --- | --- | --- | --- | --- | --- | --- | --- | --- | --- | --- | --- | --- |
|  | Spontaneous birth | | | | | | C-section | | | | | |
|  | Count | Mean | Standard Deviation | Median | Minimum | Maximum | Count | Mean | Standard Deviation | Median | Minimum | Maximum |
| Cortisol (F)* | 15 | 0.021 | 0.011 | 0.019 | 0.007 | 0.036 | 15 | 0.010 | 0.006 | 0.008 | 0.003 | 0.027 |
| Cortisone (E)* | 15 | 1.531 | 0.169 | 1.523 | 1.271 | 1.908 | 15 | 1.122 | 0.214 | 1.022 | 0.852 | 1.566 |
| Ratio F/E | 15 | 0.014 | 0.007 | 0.012 | 0.005 | 0.027 | 15 | 0.010 | 0.008 | 0.008 | 0.003 | 0.033 |
| Corticosterone (B)* | 15 | 0.005 | 0.003 | 0.004 | 0.002 | 0.009 | 15 | 0.003 | 0.001 | 0.003 | 0.002 | 0.005 |
| Dehydrocorticosterone (A)* | 15 | 0.246 | 0.083 | 0.249 | 0.105 | 0.396 | 15 | 0.112 | 0.036 | 0.116 | 0.056 | 0.155 |
| Ratio B/A | 15 | 0.021 | 0.008 | 0.019 | 0.011 | 0.035 | 15 | 0.030 | 0.016 | 0.023 | 0.012 | 0.058 |
| CRH** | 15 | 18.189 | 11.516 | 12.222 | 7.337 | 40.140 | 15 | 22.280 | 12.895 | 25.015 | 6.087 | 40.618 |
| Progesterone* | 15 | 6.675 | 2.020 | 6.876 | 4.053 | 9.216 | 15 | 6.557 | 0.669 | 6.195 | 5.672 | 7.755 |
| 17OH-Progesterone* | 15 | 0.110 | 0.055 | 0.102 | 0.056 | 0.217 | 15 | 0.135 | 0.031 | 0.145 | 0.081 | 0.178 |
| 11β-HSD2 (F) turnover*** | 15 | 36.151 | 15.038 | 32.568 | 11.816 | 66.281 | 15 | 66.454 | 29.590 | 55.315 | 30.700 | 113.296 |
| 11β-HSD2 (B) turnover*** | 15 | 29.368 | 24.975 | 17.982 | 8.168 | 96.569 | 15 | 54.145 | 35.777 | 35.884 | 21.811 | 135.978 |
| 11β-HSD1 (E) turnover*** | 15 | 1.375 | 0.722 | 1.367 | 0.245 | 2.737 | 15 | 1.477 | 1.229 | 1.002 | 0.457 | 5.142 |
| 11β-HSD1 (A) turnover*** | 15 | 4.428 | 1.842 | 4.436 | 1.905 | 7.859 | 15 | 3.018 | 1.349 | 2.774 | 1.385 | 6.383 |
| *CRH* (mRNA) **** | 15 | 499.905 | 1448.120 | 1.617 | 0.104 | 5604.149 | 15 | 2.210 | 4.166 | 0.653 | 0.095 | 12.788 |
| *HSD11B1* (mRNA) **** | 15 | 141.286 | 210.032 | 15.930 | 1.480 | 586.730 | 15 | 42.321 | 72.142 | 7.948 | 1.395 | 230.046 |
| *HSD11B2* (mRNA) **** | 15 | 13.449 | 24.824 | 2.522 | 0.562 | 93.556 | 15 | 1.529 | 0.650 | 1.446 | 0.580 | 2.859 |

* nmol/g(tissue); ** ng/g(tissue); ***nmol/g (protein) *min ^ -1; **** ratio relative to r18S

Suppl. Table 2 Measurements of the spontaneous birth cohort by biopsy localization

|  | Spontaneous birth | | | | | | | | | | | | | | | | | |
| --- | --- | --- | --- | --- | --- | --- | --- | --- | --- | --- | --- | --- | --- | --- | --- | --- | --- | --- |
|  | Central | | | | | | Medial | | | | | | Peripheral | | | | | |
|  | Count | Mean | Standard Deviation | Median | Minimum | Maximum | Count | Mean | Standard Deviation | Median | Minimum | Maximum | Count | Mean | Standard Deviation | Median | Minimum | Maximum |
| Cortisol (F)* | 5 | 0.018 | 0.010 | 0.015 | 0.008 | 0.034 | 5 | 0.023 | 0.014 | 0.027 | 0.007 | 0.035 | 5 | 0.024 | 0.012 | 0.022 | 0.010 | 0.036 |
| Cortisone (E)* | 5 | 1.527 | 0.124 | 1.523 | 1.345 | 1.690 | 5 | 1.539 | 0.222 | 1.495 | 1.353 | 1.908 | 5 | 1.527 | 0.187 | 1.535 | 1.271 | 1.761 |
| Ratio F/E | 5 | 0.012 | 0.006 | 0.012 | 0.005 | 0.021 | 5 | 0.015 | 0.009 | 0.017 | 0.005 | 0.027 | 5 | 0.015 | 0.007 | 0.017 | 0.007 | 0.024 |
| Corticosterone (B)* | 5 | 0.005 | 0.002 | 0.004 | 0.003 | 0.009 | 5 | 0.005 | 0.003 | 0.005 | 0.002 | 0.009 | 5 | 0.005 | 0.003 | 0.003 | 0.003 | 0.009 |
| Dehydrocorticosterone (A)* | 5 | 0.234 | 0.066 | 0.243 | 0.143 | 0.325 | 5 | 0.252 | 0.098 | 0.249 | 0.123 | 0.396 | 5 | 0.253 | 0.098 | 0.253 | 0.105 | 0.372 |
| Ratio B/A | 5 | 0.020 | 0.007 | 0.019 | 0.012 | 0.026 | 5 | 0.020 | 0.005 | 0.019 | 0.012 | 0.027 | 5 | 0.022 | 0.011 | 0.024 | 0.011 | 0.035 |
| CRH** | 5 | 19.267 | 14.018 | 12.387 | 7.337 | 35.611 | 5 | 19.608 | 12.804 | 12.222 | 10.526 | 40.140 | 5 | 15.690 | 9.590 | 11.093 | 8.102 | 29.848 |
| Progesterone* | 5 | 6.908 | 2.186 | 8.047 | 4.426 | 8.729 | 5 | 6.710 | 2.471 | 6.876 | 4.053 | 9.216 | 5 | 6.406 | 1.801 | 6.305 | 4.283 | 8.794 |
| 17OH-Progesterone* | 5 | 0.112 | 0.065 | 0.111 | 0.056 | 0.217 | 5 | 0.112 | 0.056 | 0.102 | 0.062 | 0.202 | 5 | 0.105 | 0.055 | 0.097 | 0.058 | 0.197 |
| 11β-HSD2 (F) turnover*** | 5 | 39.065 | 11.856 | 38.550 | 26.728 | 53.328 | 5 | 37.819 | 19.858 | 30.460 | 17.402 | 66.281 | 5 | 31.569 | 14.705 | 32.568 | 11.816 | 51.058 |
| 11β-HSD2 (B) turnover*** | 5 | 28.823 | 19.610 | 17.864 | 11.032 | 57.761 | 5 | 26.161 | 21.365 | 17.982 | 14.409 | 64.270 | 5 | 33.120 | 36.211 | 19.202 | 8.168 | 96.569 |
| 11β-HSD1 (E) turnover*** | 5 | 1.089 | 0.649 | 0.927 | 0.245 | 2.002 | 5 | 1.528 | 0.855 | 1.559 | 0.692 | 2.737 | 5 | 1.507 | 0.721 | 1.416 | 0.760 | 2.316 |
| 11β-HSD1 (A) turnover*** | 5 | 3.571 | 1.014 | 3.634 | 1.905 | 4.591 | 5 | 4.963 | 2.028 | 4.803 | 2.008 | 7.475 | 5 | 4.749 | 2.308 | 4.436 | 2.246 | 7.859 |
| *CRH* (mRNA) **** | 5 | 224.375 | 498.903 | 1.617 | 0.384 | 1116.840 | 5 | 1124.175 | 2504.389 | 0.922 | 0.104 | 5604.149 | 5 | 151.163 | 291.108 | 1.894 | 0.320 | 667.765 |
| *HSD11B1* (mRNA) **** | 5 | 102.101 | 176.367 | 13.655 | 1.571 | 412.178 | 5 | 100.911 | 152.173 | 41.036 | 1.480 | 368.132 | 5 | 220.845 | 297.095 | 9.124 | 1.636 | 586.730 |
| *HSD11B2* (mRNA) **** | 5 | 7.698 | 10.422 | 2.928 | 0.606 | 25.769 | 5 | 21.663 | 40.441 | 1.439 | 0.562 | 93.556 | 5 | 10.986 | 16.716 | 2.522 | 0.668 | 40.192 |

* nmol/g(tissue); ** ng/g(tissue); ***nmol/g (protein) *min ^ -1; **** ratio relative to r18S

Suppl. Table 3 Measurements of the C-section cohort by biopsy localization

|  | C-section | | | | | | | | | | | | | | | | | |
| --- | --- | --- | --- | --- | --- | --- | --- | --- | --- | --- | --- | --- | --- | --- | --- | --- | --- | --- |
|  | Central | | | | | | Medial | | | | | | Peripheral | | | | | |
|  | Count | Mean | Standard Deviation | Median | Minimum | Maximum | Count | Mean | Standard Deviation | Median | Minimum | Maximum | Count | Mean | Standard Deviation | Median | Minimum | Maximum |
| Cortisol (F)* | 5 | 0.011 | 0.009 | 0.008 | 0.004 | 0.027 | 5 | 0.009 | 0.004 | 0.008 | 0.003 | 0.015 | 5 | 0.009 | 0.004 | 0.009 | 0.005 | 0.014 |
| Cortisone (E)* | 5 | 1.130 | 0.279 | 1.016 | 0.852 | 1.566 | 5 | 1.126 | 0.246 | 1.022 | 0.903 | 1.529 | 5 | 1.110 | 0.146 | 1.161 | 0.909 | 1.263 |
| Ratio F/E | 5 | 0.012 | 0.012 | 0.008 | 0.003 | 0.033 | 5 | 0.009 | 0.006 | 0.007 | 0.003 | 0.016 | 5 | 0.009 | 0.005 | 0.008 | 0.005 | 0.016 |
| Corticosterone (B)* | 5 | 0.003 | 0.001 | 0.003 | 0.002 | 0.004 | 5 | 0.003 | 0.001 | 0.003 | 0.002 | 0.005 | 5 | 0.003 | 0.001 | 0.003 | 0.002 | 0.003 |
| Dehydrocorticosterone (A)* | 5 | 0.108 | 0.043 | 0.116 | 0.056 | 0.153 | 5 | 0.110 | 0.038 | 0.100 | 0.065 | 0.155 | 5 | 0.117 | 0.036 | 0.132 | 0.060 | 0.149 |
| Ratio B/A | 5 | 0.034 | 0.021 | 0.031 | 0.014 | 0.058 | 5 | 0.030 | 0.017 | 0.026 | 0.012 | 0.053 | 5 | 0.025 | 0.011 | 0.022 | 0.019 | 0.045 |
| CRH** | 5 | 20.846 | 13.978 | 25.015 | 6.087 | 39.744 | 5 | 21.191 | 12.536 | 14.780 | 11.148 | 39.279 | 5 | 24.801 | 14.747 | 30.000 | 7.471 | 40.618 |
| Progesterone* | 5 | 6.616 | 0.600 | 6.646 | 5.922 | 7.269 | 5 | 6.800 | 0.745 | 6.850 | 6.055 | 7.755 | 5 | 6.256 | 0.677 | 6.182 | 5.672 | 7.398 |
| 17OH-Progesterone* | 5 | 0.130 | 0.024 | 0.144 | 0.095 | 0.152 | 5 | 0.139 | 0.037 | 0.152 | 0.089 | 0.178 | 5 | 0.136 | 0.037 | 0.153 | 0.081 | 0.169 |
| 11β-HSD2 (F) turnover*** | 5 | 70.462 | 35.724 | 60.879 | 31.917 | 113.296 | 5 | 62.174 | 26.629 | 55.315 | 30.700 | 92.502 | 5 | 66.726 | 32.190 | 49.853 | 37.110 | 112.832 |
| 11β-HSD2 (B) turnover*** | 5 | 61.730 | 44.076 | 41.971 | 27.544 | 135.978 | 5 | 54.344 | 35.084 | 35.232 | 26.039 | 109.694 | 5 | 46.361 | 34.042 | 35.050 | 21.811 | 105.857 |
| 11β-HSD1 (E) turnover*** | 5 | 1.214 | 1.053 | 0.825 | 0.513 | 3.082 | 5 | 1.945 | 1.807 | 1.328 | 0.827 | 5.142 | 5 | 1.271 | 0.707 | 1.027 | 0.457 | 2.338 |
| 11β-HSD1 (A) turnover*** | 5 | 2.265 | 0.697 | 2.061 | 1.733 | 3.486 | 5 | 3.592 | 1.817 | 3.278 | 1.483 | 6.383 | 5 | 3.197 | 1.193 | 3.632 | 1.385 | 4.421 |
| *CRH* (mRNA) **** | 5 | 0.730 | 0.807 | 0.468 | 0.095 | 2.123 | 5 | 2.815 | 5.145 | 0.584 | 0.195 | 11.999 | 5 | 3.085 | 5.427 | 0.703 | 0.407 | 12.788 |
| *HSD11B1* (mRNA) **** | 5 | 11.144 | 7.306 | 7.935 | 3.095 | 20.379 | 5 | 83.098 | 109.157 | 8.730 | 1.395 | 230.046 | 5 | 32.722 | 53.275 | 7.948 | 7.244 | 127.917 |
| *HSD11B2* (mRNA) **** | 5 | 1.728 | 0.715 | 1.755 | 0.994 | 2.587 | 5 | 1.336 | 0.475 | 1.446 | 0.580 | 1.788 | 5 | 1.523 | 0.803 | 1.221 | 0.825 | 2.859 |

* nmol/g(tissue); ** ng/g(tissue); ***nmol/g (protein) *min ^ -1; **** ratio relative to r18S

Suppl. Table 4 Measurements by sex of newborn

|  | sex of newborn | | | | | | | | | | | |
| --- | --- | --- | --- | --- | --- | --- | --- | --- | --- | --- | --- | --- |
|  | Female | | | | | | Male | | | | | |
|  | Count | Mean | Standard Deviation | Median | Minimum | Maximum | Count | Mean | Standard Deviation | Median | Minimum | Maximum |
| Cortisol (F)* | 15 | 0.011 | 0.008 | 0.009 | 0.003 | 0.035 | 15 | 0.020 | 0.012 | 0.015 | 0.006 | 0.036 |
| Cortisone (E)* | 15 | 1.355 | 0.213 | 1.385 | 0.999 | 1.634 | 15 | 1.298 | 0.341 | 1.232 | 0.852 | 1.908 |
| Ratio F/E | 15 | 0.009 | 0.006 | 0.007 | 0.003 | 0.027 | 15 | 0.015 | 0.008 | 0.016 | 0.005 | 0.033 |
| Corticosterone (B)* | 15 | 0.003 | 0.001 | 0.003 | 0.002 | 0.005 | 15 | 0.005 | 0.003 | 0.004 | 0.003 | 0.009 |
| Dehydrocorticosterone (A)* | 15 | 0.180 | 0.063 | 0.153 | 0.105 | 0.300 | 15 | 0.178 | 0.118 | 0.139 | 0.056 | 0.396 |
| Ratio B/A | 15 | 0.016 | 0.005 | 0.014 | 0.011 | 0.029 | 15 | 0.034 | 0.013 | 0.027 | 0.022 | 0.058 |
| CRH** | 15 | 22.342 | 12.335 | 24.243 | 6.087 | 40.618 | 15 | 18.126 | 12.092 | 11.483 | 7.729 | 39.744 |
| Progesterone* | 15 | 7.171 | 1.552 | 7.398 | 4.351 | 9.216 | 15 | 6.061 | 1.212 | 6.071 | 4.053 | 8.729 |
| 17OH-Progesterone* | 15 | 0.107 | 0.035 | 0.102 | 0.056 | 0.178 | 15 | 0.138 | 0.051 | 0.152 | 0.058 | 0.217 |
| 11β-HSD2 (F) turnover*** | 15 | 49.423 | 26.269 | 45.598 | 11.816 | 102.078 | 15 | 53.182 | 29.933 | 48.491 | 17.402 | 113.296 |
| 11β-HSD2 (B) turnover*** | 15 | 29.831 | 18.826 | 21.811 | 8.168 | 68.569 | 15 | 53.682 | 39.667 | 35.232 | 13.734 | 135.978 |
| 11β-HSD1 (E) turnover*** | 15 | 1.195 | 0.493 | 1.002 | 0.457 | 2.316 | 15 | 1.656 | 1.296 | 1.027 | 0.245 | 5.142 |
| 11β-HSD1 (A) turnover*** | 15 | 3.087 | 1.348 | 2.774 | 1.385 | 6.221 | 15 | 4.359 | 1.896 | 4.043 | 1.483 | 7.859 |
| *CRH* (mRNA) **** | 15 | 46.272 | 171.969 | 0.887 | 0.095 | 667.765 | 15 | 455.843 | 1452.696 | 0.703 | 0.104 | 5604.149 |
| *HSD11B1* (mRNA) **** | 15 | 57.646 | 148.684 | 8.730 | 1.395 | 586.730 | 15 | 125.961 | 173.082 | 15.930 | 1.571 | 502.562 |
| *HSD11B2* (mRNA) **** | 15 | 5.193 | 10.116 | 1.788 | 0.562 | 40.192 | 15 | 9.785 | 24.065 | 1.446 | 0.580 | 93.556 |

* nmol/g(tissue); ** ng/g(tissue); ***nmol/g (protein) *min ^ -1; **** ratio relative to r18S
